# Barriers and facilitators for therapeutic drug monitoring of beta-lactams and ciprofloxacin in the ICU: a nationwide cross-sectional study

**DOI:** 10.1186/s12879-022-07587-w

**Published:** 2022-07-13

**Authors:** Tim M. J. Ewoldt, Alan Abdulla, Puck van den Broek, Nicole Hunfeld, Soma Bahmany, Anouk E. Muller, Diederik Gommers, Suzanne Polinder, Henrik Endeman, Inge Spronk, Birgit C. P. Koch

**Affiliations:** 1 Department of Intensive Care Medicine, Erasmus University Medical Center, Rotterdam, The Netherlands; 2 Department of Hospital Pharmacy, Erasmus University Medical Center, Rotterdam, The Netherlands; 3 Department of Pharmacy, Utrecht University, Utrecht, The Netherlands; 4 Department of Medical Microbiology and Infectious Diseases, Erasmus University Medical Center, Rotterdam, The Netherlands; 5 Department of Medical Microbiology, Haaglanden Medical Center, The Hague, The Netherlands; 6 Department of Public Health, Erasmus University Medical Center, Rotterdam, The Netherlands

**Keywords:** Survey, Beta-lactam antibiotics, Ciprofloxacin, Therapeutic drug monitoring, Intensive care, Barriers, Facilitators, Implementation

## Abstract

**Background:**

Recent studies demonstrated that failure of achieving pharmacodynamic targets of commonly used antibiotics is common in critically ill patients. Therapeutic drug monitoring (TDM) can contribute to optimize the exposure of beta-lactams and ciprofloxacin. While evidence for TDM of these antibiotics is growing, translation into clinical implementation remains limited. Therefore, perceived barriers and facilitators are important for implementing TDM in this population. The primary aim of this study was to identify healthcare professionals’ barriers and facilitators for the implementation of TDM of beta-lactams and ciprofloxacin in Dutch intensive care units (ICU).

**Methods:**

We conducted a nationwide cross-sectional online survey among healthcare professionals (HCPs) involved in antibiotic treatment of ICU patients. An adapted version of the Measurement Instrument for Determinants of Innovations was sent out. Items were considered barriers when ≥ 20% of participants responded with a negative answer. If ≥ 80% of the participants responded with a positive answer, the item was considered a facilitator.

**Results:**

Sixty-four HCPs completed the survey, of which 14 were from academic hospitals, 25 from general hospitals, and 25 from teaching hospitals. Most participants were hospital pharmacists (59%) or medical specialists (23%). Eleven barriers and four facilitators for implementation of TDM of beta-lactams were identified; 17 barriers for TDM of ciprofloxacin and no facilitators. The most important barriers were a lack of conclusive evidence, organizational support, and low availability of assays. Additional barriers were a lack of consensus on which specific patients to apply TDM and which pharmacodynamic targets to use. Identified facilitators for beta-lactam TDM implementation are low complexity and high task perception, combined with the perception that TDM is important to prevent side effects and to adequately treat infections. Twenty-eight percent of participants reported that flucloxacillin could be analyzed in their hospital. Assay availability of other beta-lactams and ciprofloxacin was lower (3–17%).

**Conclusion:**

Several barriers were identified that could obstruct the implementation of TDM of beta-lactams and ciprofloxacin in the ICU. In particular, education, clear guidelines, and organizational support should be considered when creating tailored implementation strategies. Finally, evidence of beneficial clinical outcomes on TDM of beta-lactams and ciprofloxacin can enhance further implementation.

**Supplementary Information:**

The online version contains supplementary material available at 10.1186/s12879-022-07587-w.

## Background

Beta-lactams and ciprofloxacin are frequently prescribed antibiotics in intensive care units (ICU) to treat severe infections [1, 2]. The standard dosing regimens of these drugs are not based on the altered pharmacokinetics in the critically ill. Failure to achieve the pharmacodynamic target (PDT) of these antibiotics is a common problem, and might result in therapeutic failure and antibiotic resistance [3–5]. In order to increase the efficacy of beta-lactams and ciprofloxacin in critically ill patients therapeutic drug monitoring (TDM) is proposed, which is individualizing dosing by measuring drug and active metabolite concentrations [6].

TDM has been increasingly used for antimicrobial drugs over the past decades [7]. TDM-guided dosing is traditionally used to monitor the toxicity and the efficacy of drugs with a small therapeutic range, such as glycopeptides and aminoglycosides. The focus has recently been extended to the use of TDM for improving the efficacy of drugs with a wider therapeutic range in critically ill patients, such as beta-lactams and ciprofloxacin [8, 9]. Although beta-lactams have broad therapeutic indices, toxic adverse effects have been described [10, 11]. The risk of serious adverse events is especially high for patients with renal impairment or a history of neurological disorders, which are prevalent in ICU patients [12]. Conversely, underexposure can lead to therapeutic failure [3]. Furthermore, high inter-individual variability of the pharmacokinetic [PK) profile of beta-lactams and ciprofloxacin has been reported in critically ill patients [13, 14]. These arguments have led to an increasing number of studies aiming at improving the efficacy of beta-lactams and ciprofloxacin for ICU patients [15–17].

TDM-guided dosing of beta-lactams and ciprofloxacin for efficacy is currently being investigated and proposed as routine care in multiple countries [18, 19]. However, the translation into clinical implementation is only sparingly reported [1, 20–22]. A recent review suggested that several barriers need to be overcome, such as assay availability, clinical evidence, and proof of cost-effectiveness, to facilitate the optimal implementation of beta-lactams TDM in critically ill patients [23]. However, no study included in this review used a systematic method to identify barriers and facilitators. The authors recommended to reach consensus regarding clear and practical targets.

In order to successfully implement TDM-guided dosing of beta-lactams and ciprofloxacin, developing an implementation strategy can contribute to good adaptation in clinical practice [24]. An important first step is to understand barriers and facilitators that influence the implementation [25, 26]. This ensures that the implementation strategy contains relevant determinants, is feasible, and is tailored to the context [27]. Therefore, the primary aim of this study was to identify the barriers and facilitators for the implementation of TDM of beta-lactams and ciprofloxacin in Dutch ICUs. The secondary aim was to assess the availability of TDM in Dutch hospitals. This study provides an opportunity to create targeted strategies for implementing TDM of beta-lactams and ciprofloxacin.

## Methods

### Study design and participants

A cross-sectional online survey was distributed to healthcare professionals (HCP) including hospital pharmacists, physician microbiologists, and intensivists. All HCPs involved in the treatment of ICU patients were eligible for inclusion. All data and methods were handled in accordance with the General Data Protection Regulation (GDPR).

### Setting

In the Netherlands, general and teaching hospitals usually co-operate with larger academic care centers. General hospitals mostly provide basic and less complicated care. Teaching hospitals perform more complex care and have access to more resources. However, in severe or particularly complicated cases, a referral to academic hospitals is provided. Nearly all hospitals in the Netherlands have an ICU, sometimes combined with a post-operative care unit (PACU) or cardiac care unit (CCU) and are all capable of providing respiratory and circulatory support.

Antibiotic teams (A-teams) are prevalent in the Netherlands. Ever since 2012, having an A-team is strongly advised by Dutch guidelines to ensure proper antibiotic prescribing practices [28]. These A-teams guard the quality of antibiotic prescription practices in hospitals and include at least a physician-microbiologist, an internist-infectiologist, and a hospital pharmacist.

### Data collection

The online survey was programmed and distributed using LimeSurvey (version 2.06, 2021, Hamburg, German). The survey invitation was distributed to the members of the Dutch association of clinical pharmacists (NVZA) and the Dutch association of critical care (NVIC). Personalized invitations were also sent via our extensive network. After three weeks, a reminder e-mail was sent to heighten the response rate. Data was collected in June 2021 and was analyzed anonymously.

### Survey

The survey consisted of 51 questions, which were asked separately for beta-lactams and ciprofloxacin. The Measurement Instrument for Determinants of Innovations (MIDI) formed the basis of our survey [25, 29]. The MIDI is an evidence-based survey for identifying factors that might influence the implementation and uptake of interventions and is used to develop tailored implementation strategies. The tool consists of 29 questions on common determinants of implementation in the healthcare setting. According to the survey regulations, MIDI items were adapted to the context of this study [29]. Twenty-five MIDI items were included in our survey, as well as one item of the Barriers and Facilitators Assessment Instrument (BFAI] [30], and twenty-five items that were developed after consultation with healthcare professionals. We did not include MIDI items 11 and 12 because they describe the expectations of patients. In the targeted patient population, the patients are usually sedated or too ill to be informed about this specific procedure. Furthermore, we did not include items 20 and 29 as these statements were not considered relevant.

Nineteen additional questions covered the availability of TDM in the respective hospitals, the PDT that is being used, and other potential barriers and facilitators. Four open-ended questions addressed the main important barriers and facilitators perceived by the participant. Two questions regarding the effect of the ongoing COVID-19 pandemic were also included. Most questions were scaled from 1 (“totally disagree”) to 5 (“totally agree”), and some 1 (No), 2 (Not applicable/Do not know) and 3 (Yes).

Finally, several questions were included about the participants’ characteristics (age, sex, profession, discipline, and years of experience in the current profession).

This survey was tested before distribution by a panel consisting of an intensive care specialist, a physician microbiologist, and a hospital pharmacist. The wording of some questions was altered to prevent ambiguity.

The complete survey, in Dutch and translated to English, is available in Additional file 2: Table S2.

### Statistical analysis

‘R’ (version 4.0.4, Vienna, Austria, 2021) was used for analysis with packages ‘likert’ (version 1.3.5) and ‘tidyverse’ (version 1.3.0). Descriptive statistics were reported as mean and standard deviation, or counts and percentages. Positively worded items were considered barriers when ≥ 20% of participants responded with a negative answer (“totally disagree” or “disagree”). If ≥ 80% of the participants responded with a positive answer (“agree” or “totally agree”), the item was considered a facilitator. For negatively worded items, the opposite was applied: if ≥ 80% disagreed, the statement was considered a facilitator, whereas statements to which ≥ 20% agreed were considered barriers. These cut-offs are well established among studies using the MIDI-questionnaire [31, 32]. A general inductive approach was used for analyzing the qualitative data [33].

## Results

### Participants

Eighty-nine personal invitations to fill in the survey were distributed, which resulted in 20 completed surveys (22%). Additionally, 44 surveys were completed using the distributed links in newsletters. No monetary compensation was provided for completing the survey.

Sixty-four participants completed the survey, of which 14 (22%) worked in academic hospitals, 25 (39%) in general hospitals, and 25 (39%) in teaching hospitals (Table 1). The median duration of professional work experience was 10 years (range 1–35). Most participants were hospital pharmacists (n = 38, 59%) or medical specialists (n = 15, 23%). The department of the participants was most frequently the hospital pharmacy (n = 45), followed by intensive care (n = 10) and microbiology and infectious diseases (n = 9).

**Table 1:** Characteristics of the participants

Department	Intensive care (n = 10)	MMB and infect dis (n = 9)	Hospital pharm (n = 45)	Total (n = 64)
*Age (year)*				
26–35	1 (10%)	0 (0%)	17 (37.8%)	18 (28.1%)
36–45	3 (30%)	6 (66.7%)	15 (33.3%)	24 (37.5%)
46–55	4 (40%)	3 (33.3%)	10 (22.2%)	17 (26.6%)
> 55	2 (20%)	0 (0%)	3 (6.7%)	5 (7.8%)
*Hospital beds*				
301–500	2 (20%)	1 (11.1%)	10 (22.2%)	13 (20.3%)
501–700	3 (30%)	2 (22.2%)	9 (20.0%)	14 (21.9%)
> 900	2 (20%)	2 (22.2%)	8 (17.8%)	12 (18.8%)
Missing	3 (30%)	4 (44.4%)	18 (40.0%)	25 (39.1%)
*ICU beds*				
9–16	4 (40%)	3 (33.3%)	16 (35.6%)	23 (35.9%)
17–23	2 (20%)	0 (0%)	8 (17.8%)	10 (15.6%)
24–30	1 (10%)	2 (22.2%)	4 (8.9%)	7 (10.9%)
> 30	1 (10%)	2 (22.2%)	7 (15.6%)	10 (15.6%)
Missing	2 (20%)	2 (22.2%)	10 (22.2%)	14 (21.9%)
*Type of hospital*				
Academic	2 (20%)	4 (44.4%)	8 (17.8%)	14 (21.9%)
General	4 (40%)	3 (33.3%)	18 (40.0%)	25 (39.1%)
Teaching	4 (40%)	2 (22.2%)	19 (42.2%)	25 (39.1%)
*Profession*				
Physician-assistant	1 (10%)	0 (0%)	0 (0%)	1 (1.6%)
Resident	0 (0%)	0 (0%)	5 (11.1%)	5 (7.8%)
Physician-microbiologist	0 (0%)	5 (55.6%)	0 (0%)	5 (7.8%)
Medical specialist	8 (80%)	4 (44.4%)	3 (6.7%)	15 (23.4%)
Hospital pharmacist	1 (10%)	0 (0%)	37 (82.2%)	38 (59.4%)
*Experience (years)*				
Mean (SD)	10.7 (8.5)	9.7 (3.9)	12 (9.10)	11.4 (8.42)
Median [Min, Max]	10.5 [1, 25]	10 [5, 15]	10.0 [1, 35]	10.0 [1, 35]
*Use of BLA TDM*				
Never	3 (30%)	3 (33.3%)	13 (28.9%)	19 (29.7%)
Rare	2 (20%)	2 (22.2%)	16 (35.6%)	20 (31.2%)
Sometimes	3 (30%)	2 (22.2%)	9 (20.0%)	14 (21.9%)
Regularly	2 (20%)	0 (0%)	3 (6.7%)	5 (7.8%)
Often	0 (0%)	2 (22.2%)	4 (8.9%)	6 (9.4%)
*Use of Ciprofloxacin TDM*				
Never	6 (60%)	5 (55.6%)	32 (71.1%)	43 (67.2%)
Rare	2 (20%)	3 (33.3%)	8 (17.8%)	13 (20.3%)
Sometimes	1 (10%)	0 (0%)	4 (8.9%)	5 (7.8%)
Regularly	0 (0%)	1 (11.1%)	1 (2.2%)	2 (3.1%)
Often	1 (10%)	0 (0%)	0 (0%)	1 (1.6%)
*BLA TDM experience*				
Beginner	2 (20.0%)	2 (22.2%)	13 (28.9%)	17 (26.6%)
Average	5 (50.0%)	2 (22.2%)	13 (28.9%)	20 (31.2%)
Advanced	1 (10.0%)	4 (44.4%)	4 (8.9%)	9 (14.1%)
Expert	1 (10.0%)	0 (0%)	6 (13.3%)	7 (10.9%)
Unknown	1 (10.0%)	1 (11.1%)	9 (20.0%)	11 (17.2%)
*Ciprofloxacin TDM experience*				
Beginner	1 (10%)	3 (33.3%)	13 (28.9%)	17 (26.6%)
Average	4 (40%)	1 (11.1%)	4 (8.9%)	9 (14.1%)
Advanced	1 (10%)	3 (33.3%)	3 (6.7%)	7 (10.9%)
Expert	1 (10%)	0 (0%)	4 (8.9%)	5 (7.8%)
Unknown	3 (30%)	2 (22.2%)	21 (46.7%)	26 (40.6%)

*TDM* therapeutic drug monitoring, *BLA* beta-lactam antibiotics, *MMB and infect dis* Medical microbiology and infectious diseases, *Hospital Pharm* Hospital Pharmacy

Only 70% ever came into contact with TDM of beta-lactams, and 31% of them indicated that this was seldom. Concerning ciprofloxacin, only 33% of the participants ever came into contact with TDM, of which 20% only seldom did.

### Barriers and facilitators

For the implementation of beta-lactams TDM, 11 barriers and 4 facilitators were identified. For the implementation of ciprofloxacin TDM, 17 barriers and no facilitators were found. All beta-lactams barriers were also ciprofloxacin barriers. Table 2 describes all identified barriers and facilitators, and is a summary of all the questions described in Additional file 1: Table S1.

**Table 2:** Identified barriers and facilitators influencing the implementation of therapeutic drug monitoring for ICU patients (n = 64)

Factors	Barriers	Beta-lactams (%)	Ciprofloxacin (%)
Procedure	I don’t have all the information and materials required to perform TDM	39	47
	I don’t have sufficient knowledge to use TDM	23	30
	I don’t have sufficient practical experience to use TDM	28	36
	For TDM, I am not aware of the activities I should perform and in which order		28
	Little experience with dose individualization TDM hinders me from using it		27
	The outcomes of using TDM are not clearly observable to me		20
Beliefs	The use of TDM does not saves costs	36	38
	I don’t believe dose individualization of TDM is cost-effective	20	22
	TDM increases my workload	30	25
	Colleagues don’t expect me to apply TDM		25
	TDM does not shorten ICU length of stay		20
Organization	There are no formal arrangements relating to the use of TDM	55	72
	There are other changes going on that influence implementation of TDM	36	39
	In my organization, no one have been designated to coordinate the process of implementing TDM	28	39
Literature	The lack of evidence on the effectiveness of TDM hinders me from using it	53	58
	The lack of evidence on the cost-effectiveness of TDM hinders me from using it	31	30
	*Facilitators*		
Procedure	The use of TDM to treat infections	92	
Beliefs	I feel it is my responsibility as a professional to use TDM	84	
	TDM is not too complex for me to use	81	
	TDM prevents side effects	81	

Data expressed as percentages representing the fraction of respondents that indicated that that the statement was a barrier or facilitator. The results of all questions are found in Additional file 1: Table S1

*TDM* therapeutic drug monitoring, *ICU* intensive care unit

### Barriers

Multiple barriers were identified for the implementation of TDM of beta-lactams and ciprofloxacin in critically ill patients (Table 2). The most important described barriers were that there were no formal agreements made by management (55% beta-lactams, 72% ciprofloxacin), followed by a lack of clear evidence of effectiveness (53% beta-lactams, 58% ciprofloxacin) and cost-effectiveness (31% beta-lactams, 30% ciprofloxacin). Furthermore, a substantial proportion of the participants indicated that they did not have all the information and materials to apply TDM for these antibiotics (39% beta-lactams, 47% ciprofloxacin).

### Facilitators

The facilitators identified for implementation of beta-lactams TDM were that TDM is not complex to carry out (81%), that they perceived TDM to be one of their tasks (84%), that they believed that beta-lactams TDM prevent side-effects (81%), and that it improves treatment of infections (91%) (Table 2). There were no facilitators identified for implementation of ciprofloxacin TDM.

Eight respondents (13%) indicated that the COVID-19 pandemic increased the requests for beta-lactams TDM, whereas six respondents (9%) claimed that this increased for ciprofloxacin. Additionally, some responders (n = 9; 14%) indicated that the implementation of beta-lactams TDM was hampered by the COVID-19 pandemic, whereas 11% (n = 7) argued that this was a case for ciprofloxacin.

### Qualitative analysis

We also asked responders what they consider the greatest benefit and the main disadvantage of TDM of beta-lactams and ciprofloxacin combined. The most often mentioned disadvantages were’the low availability of assays’ and ‘the absence of convincing evidence’ (both: n = 14; 21%). The greatest benefit was ‘the possible increased effectivity’(n = 29; 45%). The availability of assays, the costs and the complexity of sending blood samples to other laboratories were mostly named as the most important barriers for implementation. Eleven participants (17%) responded that patients with enhanced or diminished renal clearance should be considered for TDM. Eleven responded that all ICU patients should be considered, five participants indicated patients with overweight or underweight and four noted that it should be considered based on the micro-organism.

### Availability of analysis

**Fig. 1:**
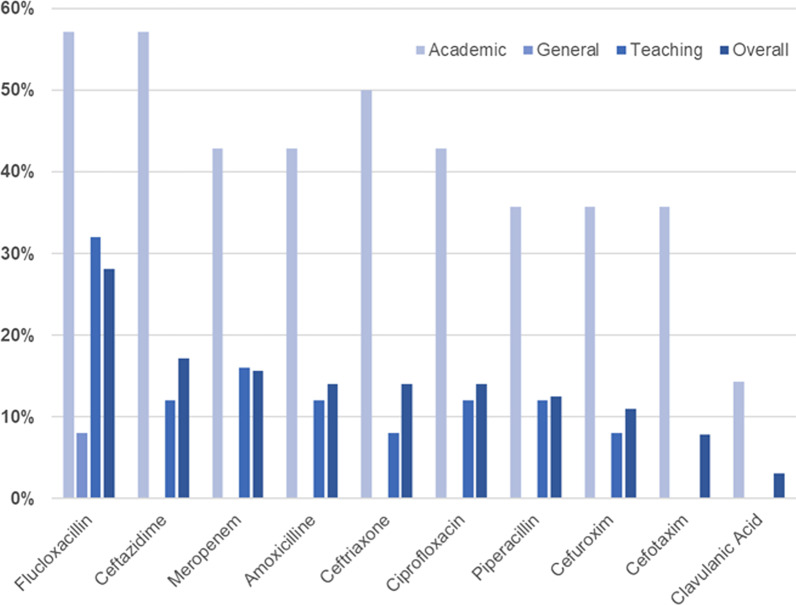
Availability of therapeutic drug monitoring of beta-lactams and ciprofloxacin

Around 28% of the participants reported that flucloxacillin could be analyzed for TDM purposes in their hospital (Fig. 1). Ciprofloxacin was available in 14% of all hospitals. Cefotaxime was the least available of the beta-lactams with only 8% availability. None of the assays were available in general hospitals, except for flucloxacillin. In academic hospitals, the availability of assays was much more prevalent, with 41% availability compared to 6% in other hospitals.

### Pharmacodynamic targets (PDT)

Of all the participants, 21 answered on possible PDT’s (Table 3). There was a great amount of variability in the answers for both beta-lactams and ciprofloxacin. For dosing TDM of beta-lactams 43% of the respondents indicate that 100% (f)T > 4xMIC should be achieved, compared to 38% that indicated that 100% (f)T > MIC should be targeted. For ciprofloxacin, 57% indicated that they did not know what target to achieve. Of the responses, 29% answered that the target of AUC/MIC > 120 should be targeted, while 14% preferred to target fAUC/MIC > 100.

**Table 3:** Reported pharmacodynamic targets for therapeutic drug monitoring of beta-lactams and ciprofloxacin in ICU patients (n = 21)

*Beta-lactams*	
50% (f)T > MIC	14%
100% (f)T > MIC	38%
100% (f)T > 4xMIC	43%
100% (f)T > MIC ECOFF	10%
Do not know	24%
*Ciprofloxacin*	
AUC/MIC > 120	29%
(f)AUC/MIC > 100	14%
(f)AUC/MIC > 90	5%
C_max_/MIC > 10	10%
fC_max_/MIC > 8	5%
Do not know	57%

*AUC* area under the curve, (f) free concentration, *T* time, *MIC* minimal inhibitory concentration, *ECOFF* EUCAST epidemiological cut-off values, *C*_*max*_ maximum concentration

## Discussion

This nationwide study provides comprehensive coverage of barriers and facilitators that influence the implementation of TDM of beta-lactams and ciprofloxacin in critically ill patients. More barriers than facilitators were identified. Barriers were mostly related to the lack of clinical evidence of TDM of beta-lactams and ciprofloxacin, lack of practical experience, low availability of assays, and no organizational support for implementation. Furthermore, only 40% of the participants had experience with beta-lactams TDM, and even fewer had experience with ciprofloxacin TDM (13%). TDM of beta-lactams was associated with a high task perception, relative ease of use, and the ability to use TDM to treat infections more effectively.

The barriers identified in our study were in line with those uncovered by Sandaradura et al. [34], who investigated barriers to the implementation of beta-lactams TDM in Australia, including lack of timely assays, a lack of training and a lack of guidelines. They also observed that the participants expected fewer clinical effects of TDM of ciprofloxacin than of other antibiotics. In the current study, we also found that ciprofloxacin TDM was less often applied and more obscure than beta-lactams TDM. Abdulla et al. reviewed current barriers and facilitators for the clinical implementation of beta-lactams TDM in critically ill patients [23]. They noted that important barriers were the limited availability of assays and a lack of guidelines, which is also reflected in our results. An ongoing study by Barreto et al. aims to identify more perspectives of HCPs in the USA on what is needed for the implementation of beta-lactams TDM using a mixed-model approach [35].

We showed that TDM of beta-lactams is not often applied, and ciprofloxacin TDM being even less often. These results are in line with other studies [21, 22, 34]. Assays for determining beta-lactams and ciprofloxacin concentrations are not yet widely available, and are mostly centered in academic hospitals. This is most likely due to the high cost and high complexity of chromatography. Mass-spectrometry (MS) has the advantage of determining a wide variability of drug concentrations but requires well-trained personnel. Continuous availability of MS is costly to implement due to the need of personnel and MS-devices. Immunoassays could be a straightforward alternative but are currently unavailable for determining beta-lactams and ciprofloxacin concentrations. Another option is the transport of patient material towards a laboratory that can analyze these drugs, however requires a strong and fast infrastructure.

Although evidence on the efficacy of beta-lactams TDM with clinical outcomes is growing [36], these studies are mostly observational. To address the lack of evidence of effectiveness, a large multi-center trial is being conducted researching the efficacy of TDM of beta-lactams and ciprofloxacin in ICU patients [15]. Making clear guidelines on how to perform TDM of beta-lactams and ciprofloxacin and on which pharmacodynamic breakpoints to target are most important for implementing these procedures. Creating organizational support and organizing education are the next steps to further clear most barriers.

Identifying patients at risk for low concentrations may help to consider and implement more individualized dosing regimens using TDM [37]. Decision aids and strong cooperation between specializations such as clinicians, pharmacists, and microbiologists can help to find the optimal population for optimizing the dosing of beta-lactams and ciprofloxacin.

TDM targets were also assessed in our study. The responses indicate that there is not yet a clear consensus for what to target during TDM. Most evidence of these targets in ICU patients is from observational trials. The ONTAI trial questioned German physicians for targets for TDM [21]. They observed a great amount of variability of what to target for beta-lactams, with most answers of experienced HCP answering 100% (f)T > 4xMIC and 100% (f)T > MIC. In a study by Wong et al., a target of 100% (f)T > MIC was most prevalent [20]. These studies have a similar conclusion, as there seems to be no clear consensus on which target to aim for.

### Strengths and limitations

Using the widely-used MIDI questionnaire to identify barriers and facilitators is a strength of this study. Another strength is that this questionnaire has reached a wide range of HCP in different hospital sizes. However, a possible limitation is that due to the methodology of this study, multiple HCPs that filled in the survey may work in the same hospital. There was, however, a clear distinction between the participants concerning the hospital size, ICU size, category of the hospital, and departments. Secondly, only Dutch HCPs were included, which should be taken into consideration when applying our results in other healthcare settings. Thirdly, most respondents had little experience with TDM, which could lead to a bias in the perceived barriers and facilitators. In a more experienced population of HCPs, the barriers and facilitators might be shifted. Finally, the cutoff point of 80% in combination with the relatively small sample size may lead to some potential facilitators not being identified as such. For example, 78% indicated that there was enough personnel available in their organization for beta-lactams TDM, but this was not included in our results.

### Future research

Implementation strategies for TDM of beta-lactams and ciprofloxacin should focus on assay availability, creating clear working instructions, education of HCPs, agreement on pharmacodynamic breakpoints, and organizational support. Future research should consider repeating this questionnaire on an international level, possibly after implementation in several hospitals has been attempted.

## Conclusion

In conclusion, we identified several factors that obstruct the implementation of TDM of beta-lactams and ciprofloxacin in critically ill patients. The discussed barriers will need to be considered when implementing TDM of these antibiotics. In particular, creating clear guidelines, assay availability, HCP education, and organizational support should all be considered when creating tailored implementation strategies. Also, further quality evidence of clinical outcomes on TDM of beta-lactams and ciprofloxacin can enhance further implementation.

## Supplementary Information

**Additional file 1: Table S1.** Barriers and facilitators influencing the implementation of therapeutic drug monitoring for ICU patients (n=64).

**Additional file 2: Table S2.** Full questionnaire in Dutch and translated to English.

## Data Availability

All data generated or analysed during this study are included in this published article.

## Abbreviations

A-teams: Antibiotic teams
BFAI: Barriers and Facilitators Assessment Instrument
HCP: Health care professional
ICU: Intensive care unit
MS: Massa-spectrometry
MIDI: Measurement instrument for determinants of innovations
PDT: Pharmacodynamic target
TDM: Therapeutic drug monitoring
