# Supplementary material for: Barriers and facilitators for therapeutic drug monitoring of beta-lactams and ciprofloxacin in the ICU: a nationwide cross-sectional study
Source: BMC Infect Dis. 2022 Jul 13;22:611. doi: 10.1186/s12879-022-07587-w (PMC9277596; doi:10.1186/s12879-022-07587-w)
Supplement: Supplementary file 1 — Additional file 1: Table S1. Barriers and facilitators influencing the implementation of therapeutic drug monitoring for ICU patients (n=64). [file 12879_2022_7587_MOESM1_ESM.docx]

Supplemental Table 1: Barriers and facilitators influencing the implementation of therapeutic drug monitoring for ICU patients (n=64)

|  |  |  | **Beta-lactam antibiotics** | | | **Ciprofloxacin** | | |
| --- | --- | --- | --- | --- | --- | --- | --- | --- |
| **No.** | **Origin item** | **Questionnaire item** | **Agree/ Totally agree (%)** | **Neutral (%)** | **Disagree/ Totally disagree (%)** | **Agree/ Totally agree (%)** | **Neutral (%)** | **Disagree/ Totally disagree (%)** |
| **Intervention** | | |  |  |  |  |  |  |
| 1 | MIDI 1 | Procedural clarity: For TDM, I know the activities I should perform and in which order | 69 | 16 | 16 | 52 | 20 | 28^‡^ |
| 2 | MIDI 2 | Correctness: TDM is based on factually correct knowledge | 55 | 36 | 9 | 34 | 55 | 11 |
| 3 | MIDI 3 | Completeness: The information and materials provided to perform TDM is complete | 47 | 14 | 39^‡^ | 30 | 23 | 47^‡^ |
| 4 | MIDI 4 | Complexity: TDM is too complex for me to use* | 6 | 13 | 81^§^ | 3 | 25 | 72 |
| 5 | MIDI 5 | Compatibility: TDM is a good match for how I am used to working | 73 | 19 | 8 | 56 | 33 | 11 |
| 6 | MIDI 6 | Observability: The outcomes of using TDM are clearly observable | 27 | 59 | 14 | 14 | 66 | 20^‡^ |
| 7 | MIDI 7 | Relevance for client: I think TDM is suitable for my patients | 53 | 44 | 3 | 38 | 56 | 6 |
| **User** | | |  |  |  |  |  |  |
| 8 | BFAI 1 | TDM leaves enough space for me to make my own considerations | 72 | 27 | 2 | 61 | 38 | 2 |
| 9 | MIDI 17 | Knowledge: I know enough to use TDM | 58 | 19 | 23^‡^ | 39 | 31 | 30^‡^ |
| 10 | MIDI 17 | Knowledge: I have enough practical experience to use TDM | 61 | 11 | 28^‡^ | 44 | 20 | 36^‡^ |
| 11 | MIDI 17 | I am aware of the methods of TDM | 75 | 6 | 19 | 59 | 13 | 28^‡^ |
| 12 | MIDI 10 | Professional obligation: I feel it is my responsibility as a professional to use TDM | 84^§^ | 13 | 3 | 72 | 25 | 3 |
| 13 | MIDI 13 | Social support: I can count on adequate assistance from my colleagues when it comes to working with TDM | 73 | 20 | 6 | 61 | 31 | 8 |
| 14 | MIDI 14 | Descriptive norm: Colleagues who are deemed to use TDM do this | 50 | 33 | 17 | 38 | 44 | 19 |
| 15 | MIDI 15 | Normative beliefs: Colleagues expect me to apply TDM | 66 | 17 | 17 | 44 | 31 | 25^‡^ |
| 16 | MIDI 16 | Self-efficacy: I am able to put TDM into practice | 75 | 11 | 14 | 58 | 25 | 17 |
| 17 | MIDI 9a | Importance outcome expectations: TDM prevent antibiotic resistance | 64 | 23 | 13 | 64 | 25 | 11 |
| 18 | MIDI 9a | Importance outcome expectations: TDM treats infection | 92^§^ | 6 | 2 | 78 | 20 | 2 |
| 19 | MIDI 9a | Importance outcome expectations: TDM prevents side effects | 81^§^ | 13 | 6 | 67 | 20 | 13 |
| 20 | MIDI 9a | Importance outcome expectations: TDM saves costs | 33 | 31 | 36^‡^ | 30 | 33 | 38^‡^ |
| 21 | MIDI 9a | Importance outcome expectations: TDM improves quality of life | 59 | 27 | 14 | 52 | 34 | 14 |
| 22 | MIDI 8 | Personal benefits: TDM helps me provide better care | 73 | 19 | 8 | 53 | 34 | 13 |
| 23 | MIDI 8 | Personal drawbacks: TDM increases my workload* | 30^‡^ | 34 | 36 | 25^‡^ | 42 | 33 |
| 24 | MIDI 8 | Personal benefits: TDM leads to higher satisfaction for myself about the treatment of the patient | 67 | 23 | 9 | 50 | 34 | 16 |
| 25 | MIDI 8 | Personal benefits: TDM costs too much time* | 13 | 31 | 56 | 13 | 39 | 48 |
| 26 | MIDI 8 | Personal benefits: TDM costs too much money* | 14 | 42 | 44 | 14 | 47 | 39 |
| 27 | MIDI 9b | Outcome expectations: TDM increases quality of care | 75 | 19 | 6 | 55 | 36 | 9 |
| 28 | MIDI 9b | Outcome expectations: TDM shortens hospital length of stay | 31 | 50 | 19 | 27 | 55 | 19 |
| 29 | MIDI 9b | Outcome expectations: TDM shortens ICU length of stay | 33 | 52 | 16 | 23 | 56 | 20^‡^ |
| **User: Organizational determinants related to TDM** | | |  |  |  |  |  |  |
| 30† | MIDI 19 | Formal ratification by management: there are formal arrangements relating the use of TDM | 38 | 8 | 55^‡^ | 16 | 13 | 72^‡^ |
| 31† | MIDI 25 | Coordinator: In my organisation, one or more people have been designated to coordinate the process of implementing TDM | 64 | 8 | 28^‡^ | 48 | 13 | 39^‡^ |
| 32† | MIDI 26 | Unsettled organization: other changes going on that influence implementation of TDM* | 36^‡^ | 14 | 50 | 39^‡^ | 17 | 44 |
| 33† |  | Has the COVID-19 pandemic increased demand for TDM? | 13 | 8 | 80 | 9 | 9 | 81 |
| 34† |  | Is the COVID-19 pandemic making implementation of TDM more difficult? | 14 | 14 | 72 | 11 | 16 | 73 |
| 35 | MIDI 21 | Staff capacity: There is enough people in our organization to apply TDM | 78 | 17 | 5 | 70 | 25 | 5 |
| 36 | MIDI 22 | Financial resources: There are enough financial resources available to use TDM as intended. | 67 | 23 | 9 | 59 | 31 | 9 |
| 37 | MIDI 23 | Time available: Our organisation provides me with enough time to include TDM as intended in my day-to-day work | 64 | 23 | 13 | 56 | 31 | 13 |
| 38 | MIDI 24 | Material resources and facilities: Our organisation provides me with enough materials and other resources or facilities necessary for the use of TDM as intended | 66 | 22 | 13 | 56 | 31 | 13 |
| 39 | MIDI 27 | Information accessible: Easy access to information about applying TDM | 67 | 25 | 8 | 59 | 31 | 9 |
| 40 | MIDI 28 | Performance feedback: In my organisation, feedback is regularly provided about progress with the implementation of TDM | 42 | 47 | 11 | 34 | 53 | 13 |
| 41 |  | The lack of evidence on the effectiveness of TDM hinders me from using it* | 53^‡^ | 23 | 23 | 58^‡^ | 27 | 16 |
| 42 |  | The lack of evidence on the cost-effectiveness of TDM hinders me from using it* | 31^‡^ | 28 | 41 | 30^‡^ | 33 | 38 |
| 43 |  | I believe dose individualization TDM is effective | 63 | 30 | 8 | 44 | 44 | 13 |
| 44 |  | I believe dose individualization TDM is cost effective | 36 | 44 | 20^‡^ | 23 | 55 | 22^‡^ |
| 45 |  | Little experience with dose individualization TDM hinders me in using it* | 19 | 28 | 53 | 27^‡^ | 33 | 41 |
| 46 |  | Uncertainty whether dose-individualization by means of TDM can lead to more complications than the current standard method hinders me in using the TDM* | 13 | 28 | 59 | 14 | 31 | 55 |
| 47 |  | The dose individualization by means of TDM leads to fewer complications than the current standard method | 31 | 56 | 13 | 25 | 61 | 14 |
| 48 |  | A guideline or advice from the Dutch Association for Intensive Care (NVIC) encourages me to use TDM | 77 | 14 | 9 | 73 | 14 | 13 |
| 49 |  | A guideline or advice from the Royal Dutch Society for the Promotion of Pharmacy (KNMP) encourages me to use TDM | 72 | 16 | 13 | 70 | 16 | 14 |
| 50 |  | A guideline or advice from the Dutch Society for Medical Microbiology encourages me to use TDM | 70 | 16 | 14 | 67 | 17 | 16 |

Data are shown as percentages.

* Indicates a negative statement. † Indicates questions with yes/no/do not know as options
‡ Totally disagree/disagree value ≥20% indicates that the determinant or item is a barrier to the implementation
§ Agree/totally agree value ≥80% indicates that the determinant or item is a facilitator for the implementation

Abbreviations: TDM: therapeutic drug monitoring, BLA: beta-lactam antibiotics, COVID-19: Coronavirus disease 2019.
