# Supplementary material for: Barriers and facilitators for therapeutic drug monitoring of beta-lactams and ciprofloxacin in the ICU: a nationwide cross-sectional study
Source: BMC Infect Dis. 2022 Jul 13;22:611. doi: 10.1186/s12879-022-07587-w (PMC9277596; doi:10.1186/s12879-022-07587-w)
Supplement: Supplementary file 2 — Additional file 2: Table S2. Full questionnaire in Dutch and translated to English. [file 12879_2022_7587_MOESM2_ESM.docx]

Supplemental Table 2: Full questionnaire in Dutch and translated to English

| **Achtergrond informatie** | **Background information** |
| --- | --- |
| Wat is uw geslacht? | What is your sex? |
| Wat is uw leeftijd? | What is your age? |
| Wat is het aantal bedden van uw ziekenhuis? | How many beds does your hospital have? |
| Wat is het aantal IC bedden van uw ziekenhuis? | How many ICU beds does your hospital have? |
| In welk type ziekenhuis werkt u? | What type of hospital do you work in? |
| Doet uw ziekenhuis mee aan de Dolphin studie? | Does your hospital participate in the Dolphin trial? |
| Wat is uw functie? | What is your position? |
| Op welke afdeling bent u werkzaam? | What department do you work in? |
| Aantal jaren ervaring in dit specialisme: | Amount of years in this specialism: |
| **Vragen over het gebruik van ‘Therapeutic Drug Monitoring’ van Beta-lactam antibiotica en ciprofloxacine** | **Questions about use of Therapeutic Drug Monitoring of Beta-lactams and ciprofloxacin** |
| Hoe vaak komt u in contact met TDM op de IC? | How often do you come into contact with TDM in the ICU? |
| Wat is uw ervaring met TDM? | How experienced are you in performing TDM? |
| Het is mij duidelijk welke stappen/activiteiten ik in welke volgorde moet uitvoeren voor TDM. | Procedural clarity: It is clear what activities I should perform and in which order for TDM |
| TDM is gebaseerd op feitelijk juiste kennis. | Correctness: TDM is based on factually correct knowledge |
| Ik heb alle informatie en materialen die nodig zijn om TDM toe te passen. | Completeness: I have all The information and materials to perform TDM |
| TDM is te ingewikkeld voor mij om te kunnen gebruiken. | Complexity: TDM is too complex for me to use* |
| TDM sluit goed aan bij hoe ik gewend ben om te werken | Compatibility: TDM is a good match for how I am used to working |
| Ik vind de effecten van het gebruik van TDM duidelijk zichtbaar. | Observability: The outcomes of using TDM are clearly observable |
| Ik vind TDM geschikt voor mijn patiënten. | Relevance for client: I think TDM is suitable for my patients |
| TDM laat genoeg ruimte voor mij om zelf afwegingen te maken. | TDM leaves enough space for me to make my own considerations |
| **Uw ervaring met ‘Therapeutic Drug Monitoring’ op de IC** | **Your experience with Therapeutic Drug Monitoring on the ICU** |
| Ik beschik over voldoende kennis om TDM te kunnen gebruiken. | Knowledge: I know enough to use TDM |
| Ik beschik over voldoende praktijk ervaring om TDM te kunnen gebruiken. | Knowledge: I have enough practical experience to use TDM |
| Ik ben op de hoogte van de methode van TDM. | I am aware of the methods of TDM |
| Ik vind het tot mijn functie behoren om TDM te gebruiken. | Professional obligation: I feel it is my responsibility as a professional to use TDM |
| Ik kan op voldoende hulp van mijn collega’s rekenen mocht ik die nodig hebben bij toepassing van TDM. | Social support: I can count on adequate assistance from my colleagues when it comes to working with TDM |
| Alle collega’s die geacht worden TDM te gebruiken, doen dat ook daadwerkelijk. | Descriptive norm: Colleagues who are deemed to use TDM do this |
| Collega’s van mijn eigen afdeling of een andere afdeling verwachten van mij dat ik TDM toepas. | Normative beliefs: Colleagues expect me to apply TDM |
| Ik ben in staat om TDM uit te voeren. | Self-efficacy: I am able to put TDM into practice |
| **Te bereiken doelstellingen met ‘Therapeutic Drug Monitoring’** | **Goals to be achieved with Therapeutic Drug Monitoring** |
| Voorkomen van antibioticaresistentie | Outcome expectations: TDM prevent antibiotic resistance |
| Behandelen van infectie | Outcome expectations: TDM treats infection |
| Voorkomen van bijwerkingen | Outcome expectations: TDM prevents side effects |
| Kosten besparing | Outcome expectations: TDM saves costs |
| Verbetering van kwaliteit van leven | Outcome expectations: TDM improves quality of life |
| Helpt me om betere zorg te leveren | Personal benefits: TDM helps me provide better care |
| Verhoogt mijn werkdruk | Personal benefits: TDM increases my workload |
| Leidt tot betere tevredenheid bij mijzelf over behandeling van de patiënt | Personal benefits: TDM leads to higher satisfaction for myself about the treatment of the patient |
| Kost te veel tijd | Personal benefits: TDM costs too much time |
| Kost te veel geld | Personal benefits: TDM costs too much money |
| Verhoogt de kwaliteit van zorg | Outcome expectations: TDM increases quality of care |
| Verkort de ziekenhuisopname | Outcome expectations: TDM shortens hospital length of stay |
| Verkort de IC opname | Outcome expectations: TDM shortens ICU length of stay |
| **Organisatie** | **Organization** |
| Zijn in uw organisatie formeel afspraken vastgelegd door het management over toepassing van TDM (in beleidsplannen, werkplannen en dergelijke)? | Formal ratification by management: there are formal arrangements relating the use of TDM |
| In mijn organisatie is / zijn één of meerdere personen aangewezen voor het coördineren van de invoering van TDM | Coordinator: In my organisation, one or more people have been designated to coordinate the process of implementing TDM |
| Zijn er, behalve de invoering van TDM, andere veranderingen waarmee u momenteel of binnen afzienbare tijd mee te maken heeft (reorganisatie, fusie, bezuinigingen, personeelsverloop, andere innovaties)? | Unsettled organization: other changes going on that influence implementation of TDM* |
| Heeft de COVID-19 pandemie ervoor gezorgd dat er meer vraag is naar TDM? | Has the COVID-19 pandemic increased demand for TDM? |
| Zorgt de COVID-19 pandemie ervoor dat de implementatie van TDM moeilijker is? | Is the COVID-19 pandemic making implementation of TDM more difficult? |
| In ons centrum wordt TDM van Beta-lactam antibiotica en/of ciprofloxacine toegepast | Beta-lactam and ciprofloxacin TDM is applied in our organization. |
| Wie maakt de keuze om TDM van Beta-lactam antibiotica in te zetten? | Who determines that beta-lactam and/or ciprofloxacin TDM should be applied? |
| Voor welke antibiotica is TDM beschikbaar? | For which antibiotics is TDM available? |
| Waarop wordt de dosering van Beta-lactam antibiotica gebaseerd bij TDM? | What is the dosing target for beta-lactam TDM? |
| Waarop wordt de dosering van ciprofloxacine antibiotica gebaseerd bij TDM? | What is the dosing target for ciprofloxacin TDM? |
| Er is voldoende personeel in onze organisatie om TDM toe te passen. | Staff capacity: There is enough people in our organization to apply TDM |
| Er zijn voldoende financiële middelen beschikbaar om TDM zoals bedoeld te gebruiken. | Financial resources: There are enough financial resources available to use TDM as intended. |
| Onze organisatie stelt mij voldoende tijd beschikbaar om TDM zoals bedoeld te integreren in mijn dagelijks werk. | Time available: Our organisation provides me with enough time to include TDM as intended in my day-to-day work |
| Onze organisatie stelt mij voldoende materialen en voorzieningen beschikbaar om TDM zoals bedoeld te kunnen gebruiken. | Material resources and facilities: Our organisation provides me with enough materials and other resources or facilities necessary for the use of TDM as intended |
| Ik heb in mijn organisatie makkelijk toegang tot informatie over toepassing van TDM. | Information accessible: Easy access to information about applying TDM |
| In mijn organisatie vindt regelmatig terugkoppeling plaats over toepassing van TDM. | Performance feedback: In my organisation, feedback is regularly provided about progress with the implementation of TDM |
|  |  |
| **Belemmeringen** | **Barriers** |
| Het gebrek aan bewijs over de effectiviteit van TDM hindert mij in het gebruik hiervan. | The lack of evidence on the effectiveness of TDM hinders me from using it |
| Het gebrek aan bewijs over de kosteneffectiviteit van dosis-individualisatie TDM hindert mij in het gebruik ervan. | The lack of evidence on the cost-effectiveness of dose-individualization TDM hinders me from using it |
| Ik geloof dat dosis-individualisatie TDM effectief is. | I believe dose individualization TDM is effective |
| Ik geloof dat dosis-individualisatie TDM kosteneffectief is. | I believe dose individualization TDM is cost effective |
| Weinig ervaring met dosis-individualisatie TDM hindert mij in het gebruik ervan. | Little experience with dose individualization TDM hinders me in using it |
| Onzekerheid of de dosis-individualisatie door middel van TDM tot meer complicaties kan leiden dan de huidige standaard methode hindert mij in gebruik van de TDM. | Uncertainty whether dose-individualization by means of TDM can lead to more complications than the current standard method hinders me in using the TDM |
| De dosis-individualisatie door middel van TDM leidt tot minder complicaties dan de huidige standaard methode. | The dose individualization by means of TDM leads to fewer complications than the current standard method |
| Hoeveel uur na afname zult u geen dosisaanpassing doorvoeren bij het inzetten van TDM? | How many hours after antibiotic administration, will you not make a dose adjustment? |
| Een richtlijn of advies van Nederlandse Vereniging voor Intensive Care stimuleert mij in gebruik van TDM. | A guideline or advice from the Dutch Association for Intensive Care (NVIC) encourages me to use TDM |
| Een richtlijn of advies van Koninklijke Nederlandse Maatschappij ter bevordering der Pharmacie stimuleert mij in gebruik van TDM. | A guideline or advice from the Royal Dutch Society for the Promotion of Pharmacy (KNMP) encourages me to use TDM |
| Een richtlijn of advies van Nederlandse Vereniging voor Medische Microbiologie stimuleert mij in gebruik van TDM. | A guideline or advice from the Dutch Society for Medical Microbiology encourages me to use TDM |
| **Andere belemmeringen** | **Other barriers** |
| Zijn er nog andere factoren die u als belemmerend ervaart voor toepassing van 'Therapeutic Drug Monitoring' van Beta-lactam antibiotica en/of ciprofloxacine op de IC? | Are there any other factors that you experience as impeding the application of 'Therapeutic Drug Monitoring' of Beta-lactam antibiotics and/or ciprofloxacin in the ICU? |
| Wat is het grootste voordeel van TDM van Beta-lactam antibiotica en/of ciprofloxacine bij de behandeling van infecties op de IC? | What is the greatest benefit of TDM from Beta-lactam antibiotics and/or ciprofloxacin in the treatment of infections in the ICU? |
| Wat is het grootste nadeel van TDM van Beta-lactam antibiotica en/of ciprofloxacine bij de behandeling van infecties op de IC? | What is the biggest disadvantage of TDM from Beta-lactam antibiotics and/or ciprofloxacin in the treatment of infections in the ICU? |
| Voor welke patiëntgroepen zou u TDM van Beta-lactam antibiotica en/of ciprofloxacine willen inzetten? | For what patient group would you like to use TDM from beta-lactam antibiotics and/or ciprofloxacin? |
